# A Bionic Self-Assembly Hydrogel Constructed by Peptides With Favorable Biosecurity, Rapid Hemostasis and Antibacterial Property for Wound Healing

**DOI:** 10.3389/fbioe.2022.901534

**Published:** 2022-06-30

**Authors:** Yang Wang, Xiao Li, Juzheng Yuan, Xudan Wang, Kaishan Tao, Jin Yan

**Affiliations:** ^1^ National and Local Joint Engineering Research Center of Biodiagnosis and Biotherapy, The Second Affiliated Hospital of Xi’an Jiaotong University, Xi’an, China; ^2^ Department of Tumor and Immunology in Precision Medical Institute, Western China Science and Technology Innovation Port, Xi’an, China; ^3^ Department of Hepatobiliary Surgery, Xijing Hospital, The Fourth Military Medical University, Xi’an, China

**Keywords:** peptide, hydrogel, self-assembly, hemostasis, antibacterial

## Abstract

Bionic self-assembly hydrogel derived by peptide as an effective biomedical hemostatic agent has always gained great attention. However, developing hydrogels with eminent-biosecurity, rapidly hemostatic and bactericidal function remains a critical challenge. Hence, we designed an injectable hydrogel with hemostatic and bactericidal function based on Bionic Self-Assembling Peptide (BSAP) in this study. BSAP was formed with two functionalized peptides containing (RADA)_4_ motif and possessed the ability to self-assemble into nanofibers. As expected, BSAP could rapidly co-assemble into hydrogel network structure *in situ* driven by Ca^2+^. The hydrogel with a concentration of 5% showed a superior microporous structure and excellent shear thinning characteristics, as well as injectability. Moreover, in the foot trauma model and tail amputation model, the fabricated hydrogel exhibited a lower blood clotting index and dramatically reduced blood clotting time and bleeding volume. Remarkably, the hydrogel reduced inflammatory responses by blocking bacterial infection, promoting wound healing. Finally, the hydrogel is highly hemocompatible and has no cytotoxicity. Overall, this work provides a strategy for developing a high-biosecurity hydrogel with hemostatic and antibacterial properties, which will allow for the clinical application of BSAP.

## 1 Introduction

Both hemorrhage and infection are common problems in traumatism and surgery, and in some cases it is even to be fatal. (Boateng and Catanzano, 2015; Sarhan et al., 2016). The excessive loss of blood at the wound site is the underlying cause of quick death. Thus, rapid and effective hemostasis should be achieved as soon as possible (Kim et al., 2019). Moreover, wound infection is the second challenge for injured patient survival, which can trigger a series of undesirable symptoms, including but not limited to pyohemia, suppurative ulceration, and septic shock (Malone et al., 2017; Sakr et al., 2018). Therefore, developing an effective coagulation and anti-infection strategy is urgently needed to rescue patients suffering from wound or trauma, especially sufferer with the coagulation disorders and poor immunity (Mofazzal Jahromi et al., 2018; Wang et al., 2020). Towards this end, although there have been numerous successful efforts using hemostatic and antibacterial biomaterials (Zhao et al., 2021), it remains a challenge to develop ideal and clinical multifunctional materials to control bleeding and prevent bacterial infection simultaneously with favorable biosecurity.

Among a large number of biomaterials, hydrogels that possess a three-dimensional polymeric network similar to natural extracellular matrix (ECM), are able to absorb and retain large amounts of water, rehydrate dead tissues and enhance self-soluble debridement (He et al., 2018; Trombino and Cassano, 2020; Ahmadian et al., 2021). Hydrogels could contribute to promoting the hemostatic plug formation and form a physical barrier at the bleeding site (Xiao et al., 2017). It also could be employed as carriers of multiple molecules, such as antimicrobial agents and growth factors (He et al., 2019; Yan et al., 2021a; Xue et al., 2022). For these reasons, hydrogels are identified as the most appropriate biomaterials for facilitating wound healing. Up to now, two broad categories of hydrogels for hemostatic and/or bactericidal materials have emerged. The first category is hydrogels made from natural polymers, such as hyaluronic acid hydrogels (Ming et al., 2021), chitosan hydrogels (Song et al., 2021), collagen (Liu et al., 2021), gelatin (Luo et al., 2019) and alginate (Li et al., 2017), which have the advantage of inherent biocompatibility. However, its allergenicity and large variations in properties between different batches restrict the widespread application of natural polymers (Echalier et al., 2017). The other category is synthetic hydrogels, such as poly (lactic acid) (Delyanee et al., 2022), polycaprolactone (Labet and Thielemans, 2009), poly (elthylene glycol) (Zhao et al., 2017), and so on. Although the controllability was improved through precise chemosynthesis, the synthetic hydrogel was still limited by the biotoxicity from the degradation of polymers (Lau and Kiick, 2015; He et al., 2022). Therefore, it is urgent to develop safe and multifunctional hydrogels which derived from nature and can be precisely synthesized.

To address these issues, natural and artificially synthesized peptide-based hydrogels have received extensive attention as hemostatic materials, especially self-assembling peptide-based hydrogels. For example, a variety of self-assembling peptides (e.g. RADA16 (Araki et al., 2021), FF8 (Shen et al., 2020), d-EAK16 (Luo et al., 2011), I3QGK (Hao et al., 2020) and so on) can aid in clotting and accelerate wound healing, which driven by non-covalent weak interaction such as hydrophobic interaction, electrostatic interactions, and hydrogen-bonding (Yan et al., 2021b). However, most peptide-based hydrogels often lacked multifunctional properties, such as appropriate mechanical properties and antibacterial activity (Hao et al., 2020; Yadav et al., 2020). Fortunately, metal ions synergism can overcome this obstacle, which are able to improve the mechanical and physically antibacterial properties of hydrogels. Most commonly, Ca^2+^ ions are introduced to promote the mechanical property of alginate hydrogels with covalently crosslinked and ionically crosslinked double-networks (Kondaveeti et al., 2018; Zhang et al., 2019). Meanwhile, Ca^2+^ ions also can accelerate thrombosis by facilitating the conversion of prothrombin into thrombin (Mahaut-Smith, 2013). As previous studies have reported, Ce^3+^ ions have been used as antibacterial agents in medicine for a long time due to the high safety and broad range of antibacterial activity. Benefit from electron-hole separation and the production of reactive oxygen species, Ce^3+^ ions are able to prevent infection during the inflammation phase of wound healing (Lansdown et al., 2003; Zamani et al., 2021). Whereas, the above metal ions also need matrix as carrier to safely perform their functions.

Herein, we designed a Bionic Self-assembling Peptide (BSAP) hydrogel with hemostasis and antibacterial property. Specifically, we firstly constructed hemostasis hydrogel based on two self-assembly peptides. In addition to the self-assembly ability, these peptides also exhibited excellent capability in response to Ca^2+^ and coagulation factor XIIIa to form a kind of hydrogel with a porous structure. Both *in vitro* and *in vivo* experiments, the BSAP hydrogel showed strong hemostatic ability, which was achieved through the aggregation and adhesion of red blood cells (RBCs) and platelets. The rare-earth element cerium encapsulated in the hydrogel and the barrier structure of hydrogel in response to the blood effectively prevented the invasion of *E. coli* and *S. aureus* to infect wound, which effectively protected wounds from purulence, decreased the leukocyte level in blood, and accelerated the wound healing. In addition, the BSAP hydrogel also had high biocompatibility without any cytotoxic effect on organs and cultured cells. In brief, this study provided an effective strategy to contruct a bionic self-assembly polypeptide hydrogel with favorable biosecurity, rapid hemostasis and antibacterial property for wound healing.

## 2 Materials and Methods

### 2.1 Preparation of Bionic Self-Assembling Peptide (BSAP)

Two self-assembling peptide sequence: Enzyme-reaction chain, (ERC, RADARADARADARADA-GGQQLK) and calcium binding chain (CBC, RADARADARADARADA-GSVLGYIQIR) were synthesized using the optimized HBTU activation/DIEA *in situ* neutralization protocol developed by an HBTU/HOBt protocol for FMOC-chemistry SPPS by employing best suited resins using the CS bio 336X automated peptide synthesizer. The detailed synthesis and purification procedures have been given in previous reports (Yan et al., 2021b). To prepare the BSAP hydrogel, the synthesized ERC and CBC with a ratio of 3:1 were dissolved in deionized water containing 0.1 mg/ml CaCl_2_ and 0.4 mg/ml Ce(NO_3_)_3_ (pH 7.0) at required concentration under room temperature.

### 2.2 Gelatinization Analysis

The hydrogels of various concentrations (0–1, 2.5, 5 and 7.5%) were prepared to explore the appropriate concentration. The gel densest was represented by the absorbance at 600 nm detected by a micro-plate reader (Epoch, BioTeK). For the cross-link process, we soaked the BSAP hydrogel in FXIIIa (Solarbio, 10U/mL in PBS) and incubated at 37°C for 12 h to achieve better cross-linking effect.

### 2.3 Morphological Characterization

The microscopic morphology of BSAP was observed using scanning electron microscopy (SEM) using a Hitachi S-4800 scanning electron microscope, after spraying the lyophilized BSAP with ∼8 nm platinum layer. The element analysis was performed using an EDX measurement incorporated by SEM. In addition, transmission Electron Microscopy (TEM, Tecnai G2 Spirit, FEI) was used for observing the microstructure of BSAP hydrogel. The samples were prepared by dripping 10 μL of BSAP solution on a copper grid coated with carbon film. Then the samples were observed after staining with 2% phosphotungstic acid solution.

### 2.4 Blood Cell Adsorption and Morphology

In order to study the blood cell adsorption ability of BSAP hydrogel. The BSAP hydrogel was mixed with whole blood and incubated for 5 minutes in a 37°C constant temperature incubator. The acquired samples were washed 3–5 times with PBS after the constant temperature incubation. Then the 3% glutaraldehyde was used to fix the samples for 3 h at 4°C. After twice dehydrating with 30, 50, 70, 80, 90, 95 and 100% ethanol, samples were freeze-dried for further morphological observation.

### 2.5 Rheological Analysis

The rheological properties of BSAP hydrogel were measured using a Discovery HR-2 hybrid rheometer from TA Instruments. All measurements were performed with a 40 mm parallel plate with a 1,000 μm gap size at 25°C. To assess the shear-thinning behavior of the hydrogel, viscosity was recorded as a function of shear rate, which was ramped from 0.01 to 100 s. To assess the self-healing behavior, the G′ and G″ were measured by three alternate step-strain cycles (1% for 60s and 1,000% for 60 s).

### 2.6 Blood Clotting Index Assay

The pro-coagulant effect of the BSAP hydrogel was assessed by measuring the blood clotting index (BCI) according to a previously reported protocol (Gaharwar et al., 2014). Briefly, recalcified whole-blood was prepared by adding the 0.2M CaCl_2_ into freshly collected sodium citrate blood. In the experimental group, 50 μL recalcified whole-blood was added into equal volume of BSAP hydrogel. After 0, 30, 60, 90, 120, 150, 180 and 210 s of incubation at 37°C, each well was washed with 5 ml deionized water and the liquid was immediately removed. The recalcified whole-blood (50 μL) was used as control and subjected to the same process. The OD of the supernatant was measured at 600 nm absorbance. The BCI were quantified as formula:

$$BCI=\frac{As-Ac}{Ar-Ac}\times 100\%$$

Where *As* represented the absorbance of samples, *Ar* represented the absorbance of reference (50 μL recalcified whole-blood in 5 ml deionized water) and *Ac* represented the absorbance of deionized water.

### 2.7 Bacterial Strains

*Escherichia coli* (*E. coli,* ATCC 25922) and *Staphylococcus aureus* (*S. aureus*, ATCC 25923) used in this study were grown in LB broth overnight. The concentration of bacteria was regulated to certain concentrations by turbidimetry for *in vitro* adhesion assay and wound infection model.

### 2.8 *In vitro* Antibacterial Assay

Prior to antibacterial experiments, the BSAP samples were sterilized under UV light for 15 min. One day before the assays, the human umbilical vein endothelial cell (HUVECs) cell line, purchased from Cell Bank of Shanghai Chinese Academy of Sciences (Shanghai, China), were seeded into 24-well plates at a density of ∼10^5^ cells/well in the culture environment of 5% CO_2_ and 37°C. Morrow, the BSAP was add on the upward side of the cells in the experimental group. Then bacteria (*E. coli* or *S. aureus*) from mid-exponential phase was add to the cells at a multiplicity of infection of 100 (MOI = 100). After 0, 1, 3, 5, and 7 h of incubation, supernatant and BSAP were removed and the cells were lysed with 0.1% Triton/H_2_O after gently washing to collect the living bacterial adhered to cells. The lysate was collected and diluted with LB broth for enumerating CFU, which reflects the adhesion population of bacteria on cells.

### 2.9 *In Vivo* Experiments

All experiments were conducted according to the ethical standards and protocols accepted by the Committee of Animal Experimentation guidelines approved by the Fourth Military Medical University’s medical ethics committee (IACUC-20210517). The experimental animals (200 g male Sprague-Dawley rats) were purchased from the Experimental Animal Center of the Fourth Military Medical University. The rat foot trauma model, rat tail amputation model and full-thickness skin wounds model were constructed for further experiments under general anesthesia using isoflurane (R510-22, VETEASY).

#### 2.9.1 Rat Foot Trauma and Infection Model

The anesthetized rats were placed in the prone position and fixed on a sterile operating table. Pre-weighed filter paper was placed beneath the right foot of the rats after disinfecting with 70% alcohol. To create a bleeding wound, the skin was incised with length about 1 cm longitudinally along the foot pads of the rats with a scalpel. When the rat foot pads were incised, the wound was treated with about 200 μL BSAP hydrogel immediately. The weight change of the filter paper was measured after the wound was sufficiently hemostasis and the bleeding time was recorded at the same time. In the infection group, about 1×10^10^ *E. coli* and 1 × 10^10^ *S. aureus* were added onto the wounds and the wounds treated with BASP hydrogel. After the experiment, the surface of the foot pad was covered and fixed with a medical surgical sterile dressing. The wound healing was monitored on the third day after the operation, and the foot pad skins were collected from the wound area for histological observation.

#### 2.9.2 Rat Tail Amputation Model

As mentioned above, the rat was placed in prone position and fixed on a sterile operating table after anesthesia. The entire tail of the rat was sterilized with 70% alcohol. Pre-weighed filter paper was placed beneath the tail and the end of the rat tail (∼8 cm) was transected with a scalpel. In treatment group the wound was treated with 200 μL BSAP hydrogel immediately after transection of the rat tail. Similarly, the weight gain of the filter paper was recorded after the wound was hemostasis sufficiently, and the bleeding time was recorded at the same time. The wound was covered with a sterile dressing postoperatively and observed on the third day after the operation.

#### 2.9.3 Full-Thickness Skin Wounds Infection Model

One day before the experiment, the skin surface hair was shaved from the back of the rat. During the experiment, the anesthetized rats were fixed in the prone position. The skin of the experimental site was disinfected with 70% alcohol. A circular incision with a diameter of 8 mm was made by excising the epidermis and dermis on left side of the midline of the rat’s back using a skin punch and surgical forceps. In the treatment group, 200 μL BSAP hydrogel was smeared into the wounds. In the infection group, about 1×10^10^ *E. coli* and 1 × 10^10^ *S. aureus* were added onto the wounds and the wounds treated with BASP hydrogel. The medical surgical sterile dressing was then used to cover the wound. Thereafter, the wound surface was wiped with sterile saline and the dressing changed every 3 days. In addition, the wound healing was observed and photographed at fixed time points. The blood samples were collected from the retro orbital vein of anesthetized rat first day (for blood routine examination and ELISA assay) and third day (for blood routine and blood biochemistry examination) after modeling and treatment. The rat back skin samples were collected 2 weeks after modeling treatment and fixed with 4% paraformaldehyde.

### 2.10 Blood Routine and Blood Biochemistry Examination

Whole blood was used for routine blood tests, which were assayed by an automatic blood analyzer (BC-2800vet, Mindray). Serum was separated from whole blood samples following centrifugation at 6,000 g for 10 min for blood biochemistry examination, which was assessed by an automated biochemical analyzer (Chemray 800) using the corresponding kits (Kayto) according to their instructions.

### 2.11 Enzyme-Linked Immunosorbent Assay

Serum samples for ELISA were collected on day 1 after modeling and treatment of full-thickness skin wounds infection model. The serum was subjected to ELISA for measurement of IL-6 and TNFα according to the manufacturer instruction (Jianglaibio).

### 2.12 Hematoxylin-Eosin Staining

Hematoxylin and eosin staining were performed on paraffin-embedded tissue slices (40 μm) for histological analysis. A microscope was used to capture all of the slices (IX53, Olympus, Japan).

### 2.13 Cytotoxicity Tests

*In vitro* cytotoxicity of BSAP hydrogel was evaluated using AML12 and HUVECs cell lines, which sourced from the Cell Bank of the Shanghai Chinese Academy of Sciences (Shanghai, China). Briefly, cells were seeded in a 96-well plate (1,000 cells per well) and cultured for 24 h in corresponding growth medium. Afterwards, culture media was replaced by fresh media containing BSAP hydrogels at different concentrations (0.075, 0.05, 0.025 and 0%). The matching media was replaced every day. Then the viability of the cells was measured on day 0, 1, 2, 3, 5, and seven using CCK8 reagent according to the manufacturer instruction (Dojindo). The absorbance of each well was measured using an ELISA reader at 450 nm. Further, the AML12 cells were cultured in the fresh medium and fresh medium containing with 0.075% BSAP respectively. In addition, we also seeded the AML12 cells on the 5% BSAP and cultured in the suitable medium. After 24h, 4 µM calcein-AM and 1.5 µM PI working solution was added to cells and incubated at 37°C for 45min. Then, the wells with samples were observed under a confocal laser scanning microscope Zeiss LSM 780 (Leica Microsystems, Wentzler, Germany).

### 2.14 Statistical Analysis

Each of the data were presented as means ± SD. Each experiment was repeated three times with three replications per experiment, if not otherwise specified. For statistical significance analysis, student t-test was performed via GraphPad Prism at the probabilities of *P ˂ 0.05, **P ˂ 0.01, ***P ˂ 0.001 and ****P ˂ 0.0001 to show the levels of statistically significance.

## 3 Results and Discussion

### 3.1 Characteristics of BSAP

**FIGURE 1:**
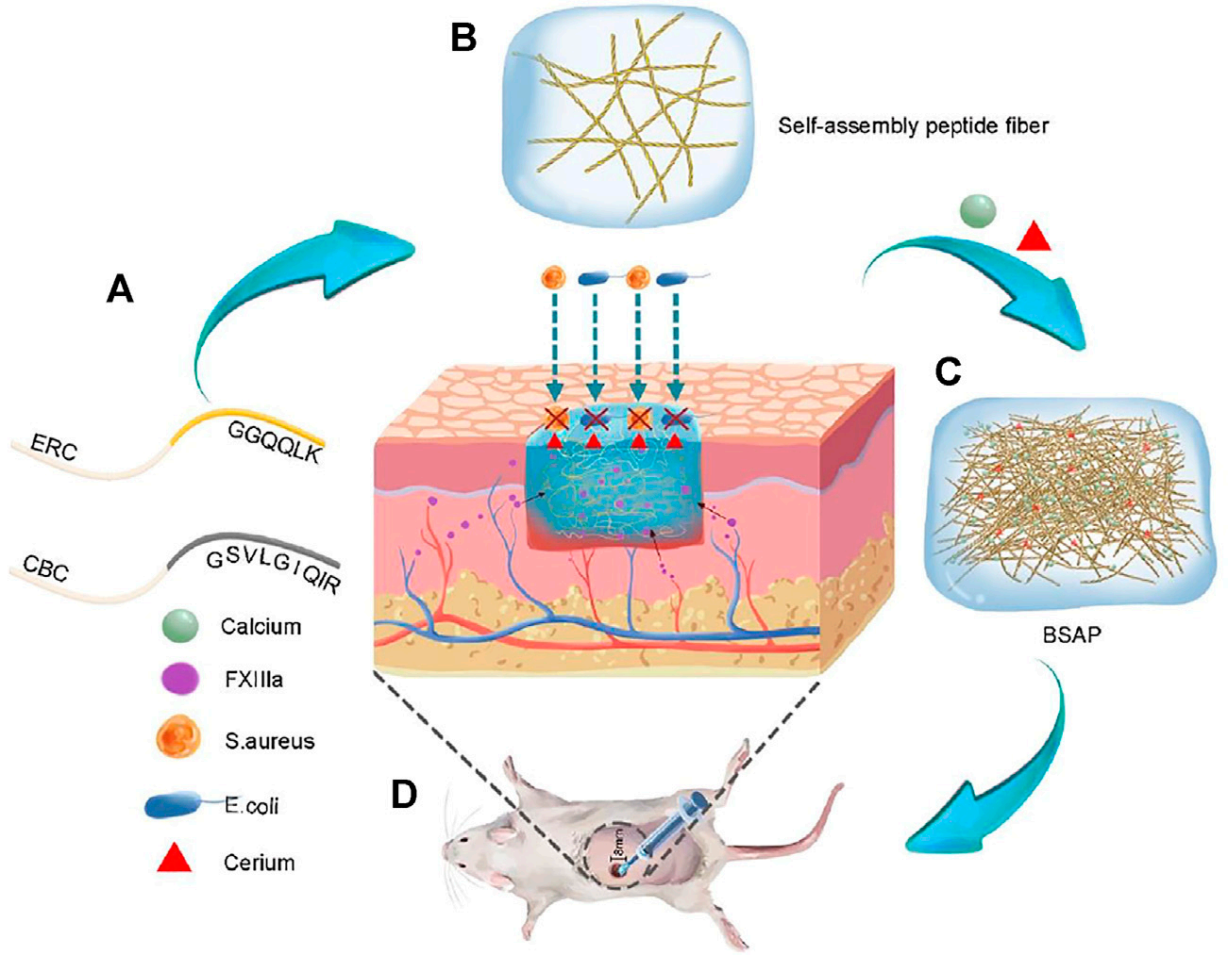
Schematic representation formation process and function of Bionic Self-Assembling Peptide (BSAP). **(A)** The structure diagram of the peptide chain: calcium banding chain (CBC) and enzyme-reaction chain (ERC). **(B)** The sparsely wired nano fibers formed by the conjugation of (RADA)_4_ backbone. **(C)** The BSAP hydrogel with tightly entangled mesh-like nanofiber network that was formed by the functional motifs of CBC in response to calcium. The calcium and rare earth element cerium were bound on the nanofiber. **(D)** The function schematic diagram of BSAP in wound: hemostasis and antibacterial.

Self-assembling hydrogels constructed by peptide have been regarded as a class of ideal material with various of biofunction, due to their inherent biocompatibility, mechanical tunability, and biochemical properties (Fichman and Gazit, 2014; An et al., 2021). Herein, a multifunctional hydrogel with the significant hemostasis and antibacterial ability was constructed by the self-assembly between two designed peptides: a Ca^2+^ binding chain termed CBC and an enzyme responding chain named ERC (Figure 1).

**FIGURE 2:**
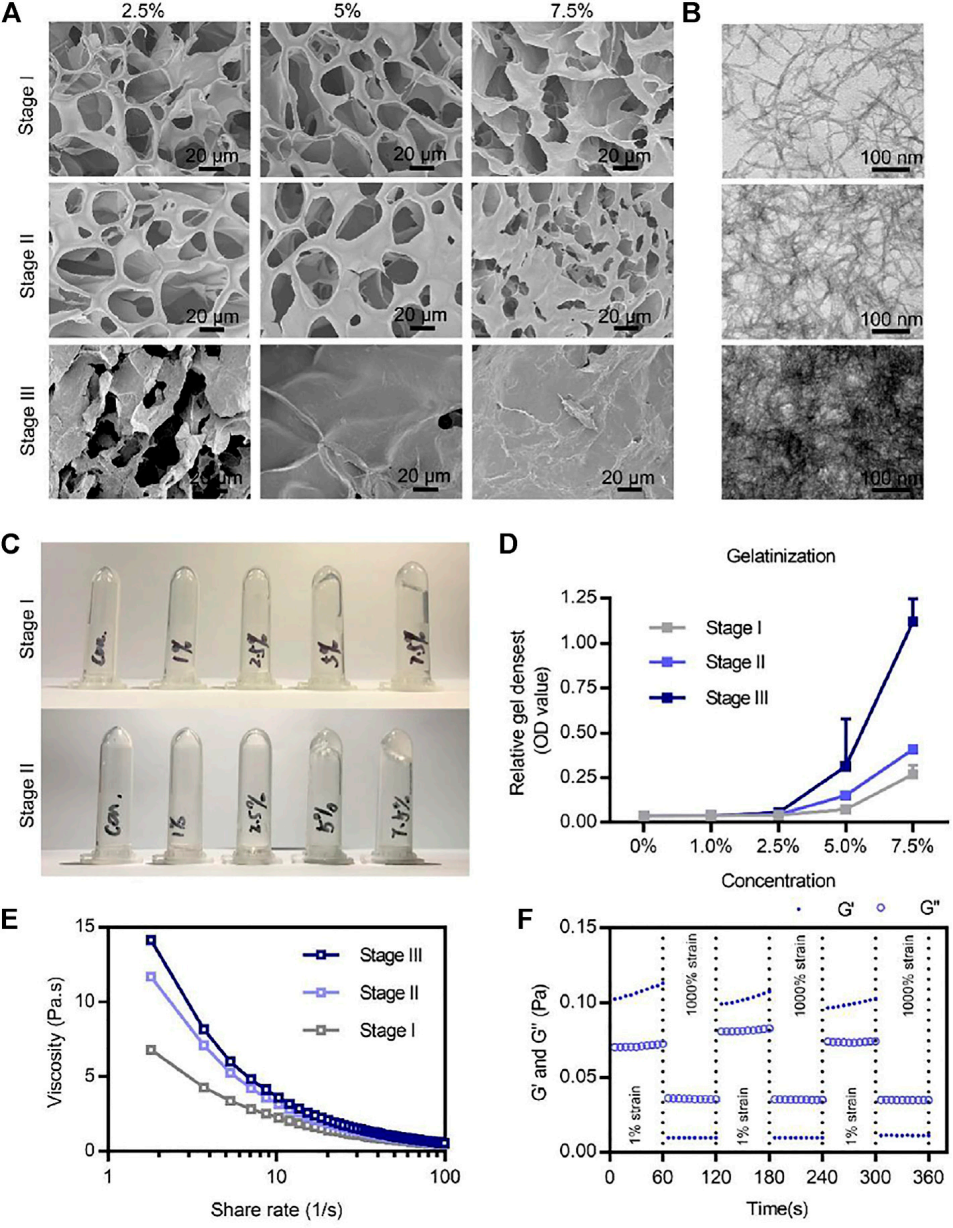
Microstructures and physicochemical properties of the BSAP hydrogel. **(A)** SEM image of the three stages’ BSAP hydrogels at different concentrations. Scale bar = 20 µm. **(B)** TEM image of nanofibers of the three stages’ BSAP hydrogels. Scale bar = 100 nm. **(C)** Gelatinization photography of BSAP hydrogels at stage I and stage II. **(D)** Gelatinization quantized at the absorbance at 600 nm (*n* = 3). **(E)** Viscosity-shear rate profiles of the BSAP hydrogel with the shear rate ranging from 0.01 to 100 s ^−1^ (*n* = 3). **(F)** The shear recovery property of the BSAP hydrogel when alternating the strain from 1 to 1,000% periodically (*n* = 3).

The formation of BSAP hydrogel underwent three main stages. In the stage I, driven by the hydrophobic and electrical interaction between RADA motifs, CBC and ERC were self-assembled into sparsely wired nanofibers, which can be observed in the SEM and TEM images (Figures 2A, B). In the stage II, the sparsely wired nanofibers further crosslinked into the tightly entangled nanofibers via the interaction between Ca^2+^ and CBC, resulting in the hydrogelation. At the same time, the adding of rare-earth element cerium (Ce^3+^) further endowed this hemostatic hydrogel with antibacterial ability (Figure 1). The introduction of calcium and cerium were characterized by elemental analysis (Supplementary Figure S2). In the stage III, the functional motif GGQQLK around the ERC fibers would further crosslink with each other and form a compact structure *via* the acyl transfer reaction mediated by coagulation factor XIIIa (Figures 2A, B).

Next, we explored the absorption value of BSAP that reflected the gel density in different concentrations at different stages. The increasing value corresponding to the stages also proved the Ca^2+^ and FXIIIa mediated crosslinking (Figure 2C). At the same time, the BSAP hydrogel exhibited a more sensitive response to calcium until the density of 5%, at which the fluid gel transferred to solid gel (Figure 2C), as well as the increased absorption value (Figure 2D). In addition, as shown in the SEM image of Figure 2A, the average pore of the hydrogel was 20 μm when the density was 5% on stage II, which was considered as suitable in adsorption and transportation of RBCs and platelets, thus it was better for hemostasis (Teng et al., 2021). Therefore, this density was used for further experiments.

To further study the mechanical stability of the BSAP hydrogel, we investigated the viscosity–shear rate profiles and shear recovery measurements of hydrogel by rheological tests. As shown in viscosity–shear rate profiles of Figures 2A, E strong shear-thinning response in BSAP hydrogel was observed, suggesting that the solid-BSAP hydrogel would transform into liquid-gel with the increased shear rate, improving the adaptability of BSAP to the various shapes of wounds. We also found an increasing viscosity under the condition of Ca^2+^ and FXIIIa, demonstrating the three-stage crosslinking. In addition, an excellent self-healing property was observed in shear and recovery assay, further suggesting the syringe ability of BSAP hydrogel. Collectively, BSAP hydrogel held a suitable structural basis for hemostasis and antibacterial.

### 3.2 Hemostatic Ability of the BSAP

**FIGURE 3:**
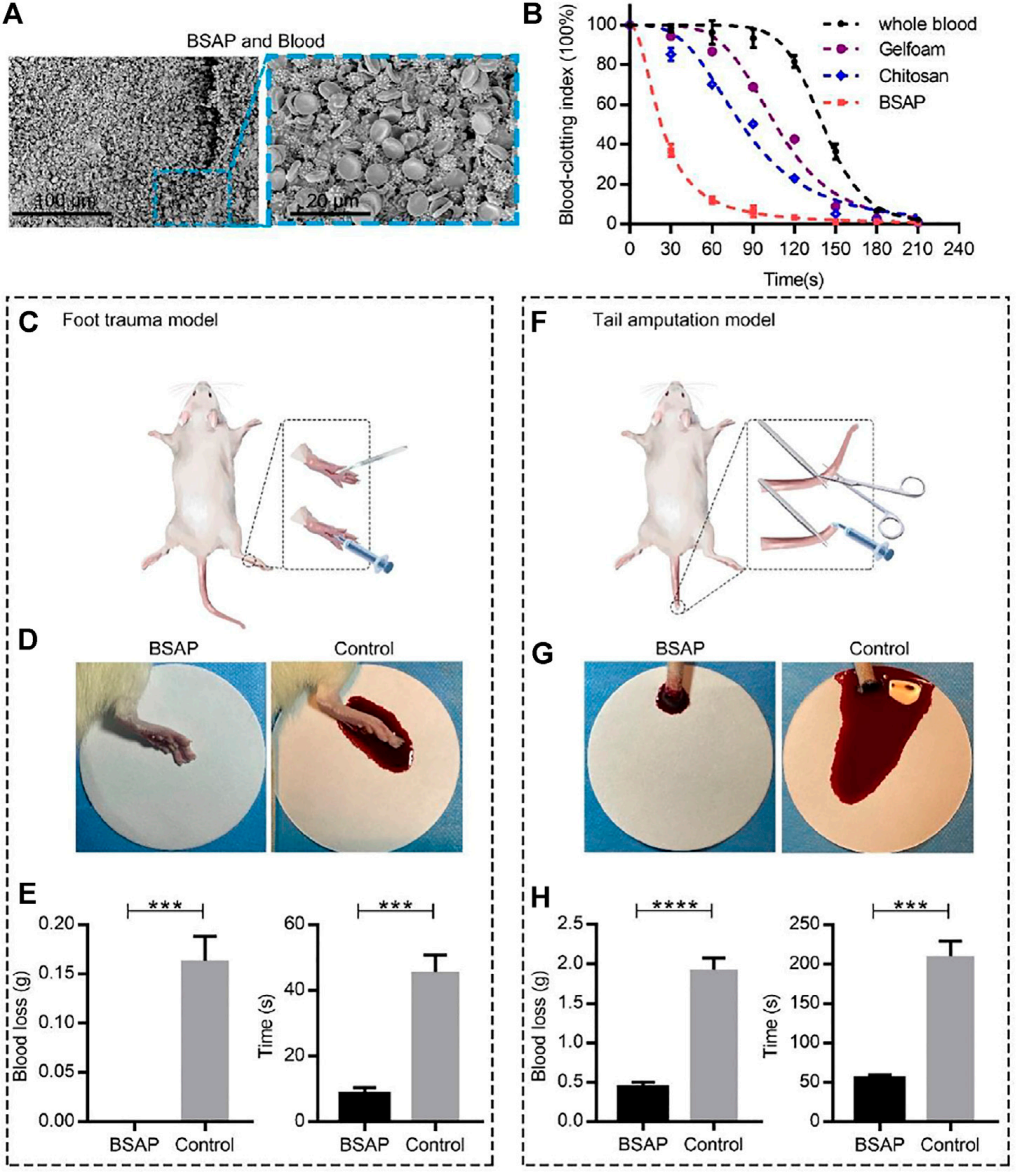
Hemostatic performance of BSAP hydrogel. **(A)** SEM of the blood clots of BSAP hydrogel integrated with whole blood. The erythrocyte and deformed platelets are gathered to form a compact structure. **(B)** The blood clotting index (BCI) of the BSAP hydrogel and controls (whole blood, Gelfoam, Chitosan) based on time (*n* = 4). The blood solutions were measured at 600 nm. **(C)** Schematic illustration of the establishment of rat foot trauma model. **(D)** Bleeding photograph and **(E)** hemostatic ability (blood loss and hemostatic time) of BSAP hydrogel in rat foot trauma model (*n* = 3, *^∗^P < 0.05, ^∗∗^p < 0.01, ^∗∗∗^p < 0.001, ^∗∗∗∗^p < 0.001*). **(F)** Schematic illustration of the establishment of rat tail amputation model. **(G)** Bleeding photograph and **(H)** hemostatic ability (blood loss and hemostatic time) of BSAP hydrogel in rat tail amputation model (*n* = 3, *^∗^P < 0.05, ^∗∗^p < 0.01, ^∗∗∗^p < 0.001, ^∗∗∗∗^p < 0.001*).

The wound healing was consisted of complex stages, among which hemostasis was the first step, which helped to prevent blood loss and benefited cell proliferation and tissue remodeling (Qu et al., 2018). To detect the hemostatic ability of the BSAP hydrogel, the dynamic blood clotting index (BCI) *in vitro* assay was performed. In this assay, the absorbance value of free hemoglobin in the supernatant of blood was measured, in which a higher absorbance value of the hemoglobin-contained super-natant indicated a slower clotting rate (Li et al., 2016; Li et al., 2018). As shown in Figure 3B, the clotting process completed at 60–90s under the condition of BSAP hydrogel in sharp contrast to the coagulation time at 180–210s of negative control (whole blood). What’s more, the BSAP also showed superior procoagulant activity over the positive controls (Gelfoam and Chitosan), indicating the excellent hemostatic effect of BSAP hydrogel. To further understand the reaction between BSAP hydrogel and the blood, BSAP hydrogel was injected into fresh whole blood, the surface morphology of the formed blood clot was observed using scanning electron microscopy (SEM). A large number of blood cells, including red blood cells (RBCs) and the deformed platelets, were adhered and gathered onto the BSAP (Figure 3A). Furthermore, a compact structure was formed after mixing with blood, which would prevent further bleeding as a compact physical barrier *in situ* at bleeding sites.

The hemostatic properties of the BSAP hydrogel were further evaluated in two rat hemorrhage models by measuring the bleeding amount and the hemostatic time: the rat foot trauma model and rat tail amputation model. Towards rat foot trauma model (Figures 3C–E), the BSAP hydrogel was immediately injected when the 1 cm length incisions were created. As shown in Figures 3D, E , compared with the control group, the hydrogel rapidly stopped the bleeding within seconds, and no blood was deposited on filter paper. After modeling 2 days, the wounds dressed with BSAP hydrogel revealed better wound repair compared to that of control group (Supplementary Figure S1). Similar results also can be observed in the rat tail amputation model, the BSAP hydrogel also reduced bleeding time and blood loss significantly, even though the rat tail amputation model showed more severe bleeding (Figures 3F–H). These results revealed that the BSAP exhibited an excellent hemostatic capacity.

### 3.3 Antibacterial Ability and Wound Healing Study of the BSAP Hydrogel

**FIGURE 4:**
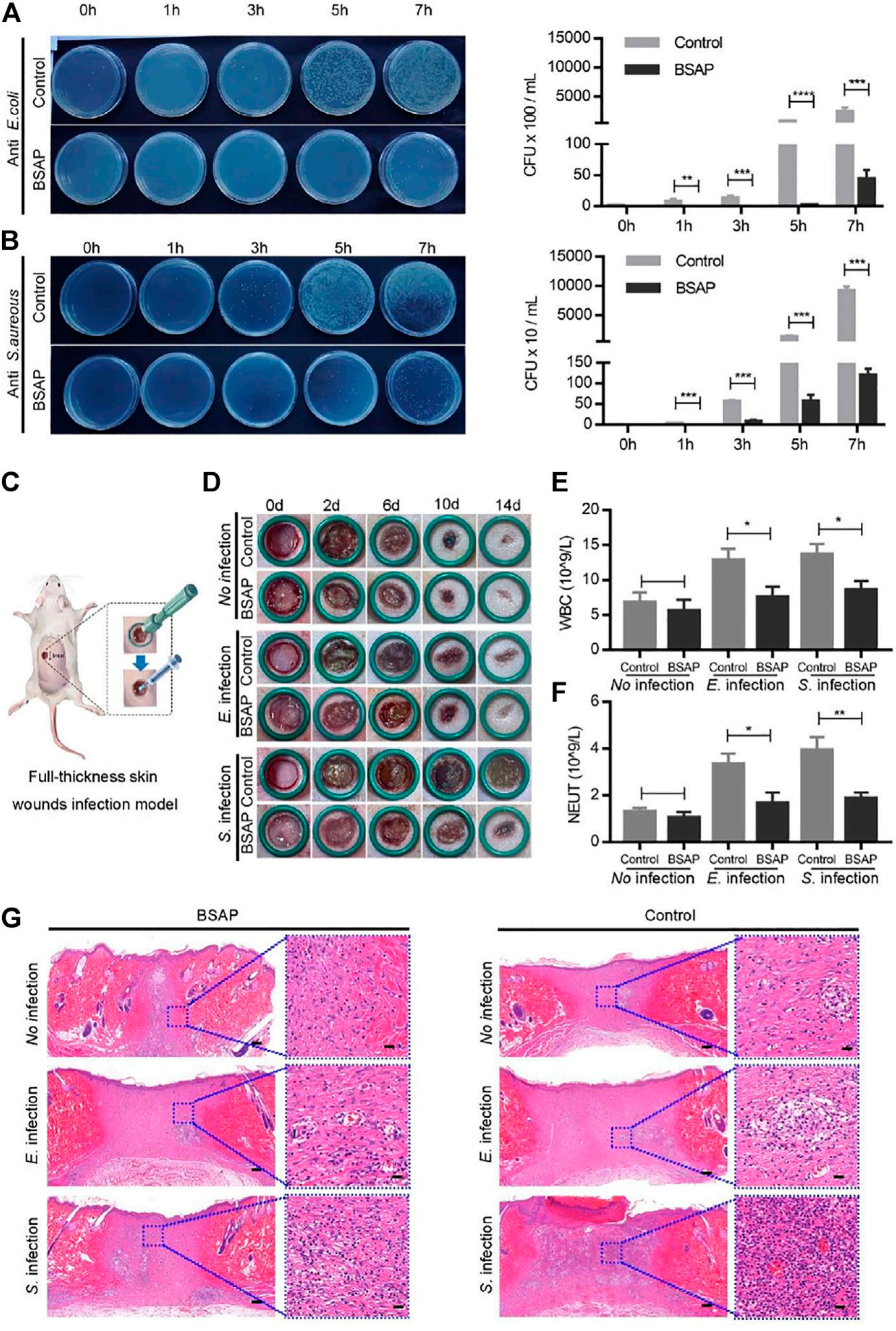
Antibacterial property of BSAP hydrogel. **(A)**. The effects of BSAP hydrogel on *E. coli* adhesion to HUVEC cells during bacterial infection. Colony counting of bacterial adhering on the cell membranes (1/1,000 dilution). (*n* = 3, *^∗^P < 0.05, ^∗∗^p < 0.01, ^∗∗∗^p < 0.001, ^∗∗∗∗^p < 0.001*). **(B)** The effects of BSAP hydrogel on *S. aureus* adhesion to HUVEC cells during bacterial infection. Colony counting of *S. aureus* adhering on the cell membranes (1/100 dilution). (*n* = 3, *^∗^P < 0.05, ^∗∗^p < 0.01, ^∗∗∗^p < 0.001, ^∗∗∗∗^p < 0.001*) **(C)** Schematic illustration of the establishment of full-thickness skin wounds infection model. **(D)** Representative wound photos infected wounds treated with BSAP hydrogel. **(E, F)** White blood cell (WBC) count **(E)** and Neutrophil (NEUT) count **(F)** of the full-thickness skin wounds infection model on day 1 after modeling treatment (n = 3, *^∗^P < 0.05, ^∗∗^p < 0.01, ^∗∗∗^p < 0.001, ^∗∗∗∗^p < 0.001*). **(G)** H and E-stained skin histological sections of wounds healing (scale bar: 200 nm) and inflammation (scale bar: 20 μm) on day 14 after modeling treatment.

Some bacteria caused wound infections and delayed the healing process (Ma and Wu, 2022). Thus, antibacterial ability, an important assessment factor for hydrogel materials, could add more value to their biological effect. Herein, the rare-earth element cerium was encapsulated to impart BSAP hydrogel with antibacterial activity. Two common bacterias, Escherichia Coli (*E. coli*) and *Staphylococcus aureus* (*S. aureus*), were used for test the antibacterial ability of BSAP hydrogel (Guo et al., 2018). *In vitro* experiments, when *E. coli* and *S. aureus* were employed to infect the HUVECs, the BSAP hydrogel was used to prevent the invasion. Then the bacteria, which adhered onto cells, at different time point were counted for evaluating the ability of BSAP hydrogel in preventing bacterial invasion. As shown in Figure 4A, a large number of *E. coli* adhered to HUVECs after 3 h incubation, while in the BSAP group, the *E. coli* was detected until 7 h incubation. A similar phenomenon was also found in *S. aureus* infection group (Figure 4B). These results demonstrated the remarkable antibacterial ability of BSAP *in vitro*.

On the basis of the *in vitro* results that the BSAP hydrogel effectively reduced the bacterial infection to cells, its effect on wound was further investigated *in vivo* using a full-thickness skin wounds infection model. Hydrogel was placed in the 8 mm × 8 mm full-thickness infection wounds on the back of rats (Figure 4C). As shown in Figure 4D, the wound site closed gradually over time, and were almost completely closed on day 14. However, the infected wounds trended a slower wound closure, especially in *S. aureus* infection group, in which some severe inflammation indications including redness and swellings were observed. In comparison, the addition of BSAP hydrogel decreased the bacteria-induced inflammatory responses as well as accelerated the speed of wound-healing significantly. The blood routine test showed that the level of white blood cells (WBCs) and neutrophile granulocyte (NEUT) decreased in BSAP groups compared to the control groups, which further demonstrated the antibacterial ability of BSAP hydrogel (Figures 4E, F and Supplementary Table S1). In addition, the results of enzyme-linked immunosorbent assay (ELISA) showed that the levels of IL-6 and TNF-α in serum decreased significantly with BSAP treatment, which further validated that BSAP blocked the infection caused by bacterial invasion effectively (Supplementary Figure S4). What’s more, on day 14, the histological images of H&E staining in BSAP treatment group showed more granulation tissue, formed epidermal tissue and hair follicle than control and infection group, while less inflammatory cells (Figure 4G), suggesting the excellent wound healing function of BASP hydrogel.

All above results indicated that the BSAP hydrogel could effectively protect wounds from bacterial infection and promote wound healing. During this process, the hydrogel acts as a physical barrier to prevent contamination and potential infection of the wound. More importantly, the antibacterial component cerium loaded in the hydrogel can kill the bacteria effectively, which may be caused by the electron-hole separation and the production of reactive oxygen species from cerium, further preventing the bacteria from attacking the wounds (Atif et al., 2019; Guo et al., 2021). This will create a suitable healing environment for the wound.

### 3.4 Toxicity Analysis of the BSAP Hydrogel

**FIGURE 5:**
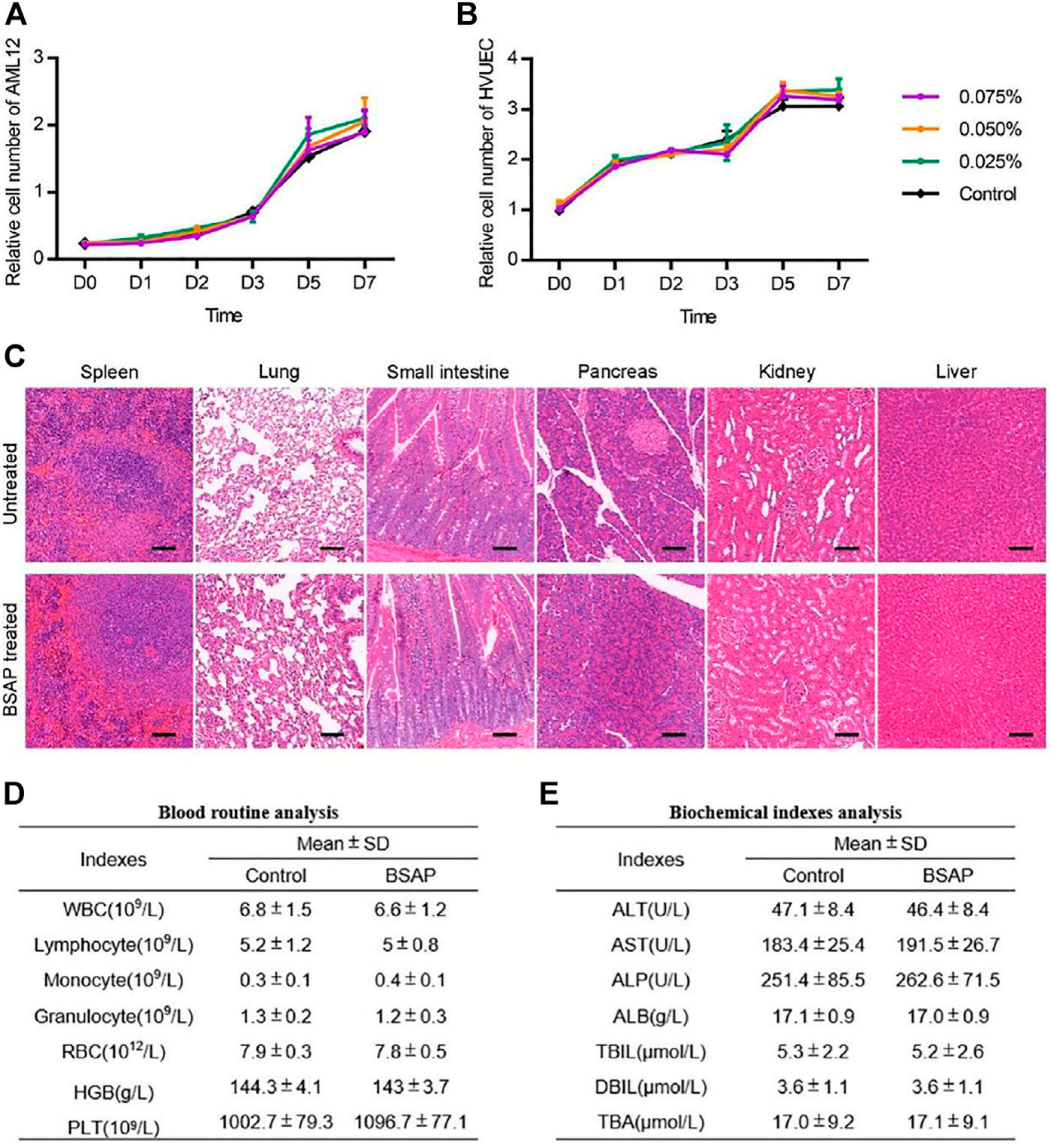
Toxicity evaluation of BSAP hydrogel. **(A)** and **(B)** Time-dependent cytotoxic activity of 0.075, 0.050, 0.025, 0% (control) BSAP in culture medium against **(A)** AML12, mouse liver cells and **(B)** HUVEC, human umbilical vein (*n* = 3). **(C)** Representative histological H and E staining images of spleen, lung, small intestine, pancreas, kidney and liver tissue sections with and without the BSAP hydrogel treatment in full-thickness skin wounds model on day 14 after BSAP hydrogel treatment. Scale bar represent 100 μm. **(D)** Blood routine analysis in full-thickness skin wounds model (without infection) on day 3 after BSAP hydrogel treatment (*n* = 3). **(E)** Biochemical index analysis in full-thickness skin wounds model (without infection) on day 3 after BSAP hydrogel treatment (*n* = 3).

Considering the potential application of the BSAP hydrogel as a clinical hemostatic agent, the material should also possess the biosecurity. Thus, we applied *in vivo* and *in vitro* experiments to assess its biological responses. The cytocompatibility of the hydrogel was first evaluated by examining the effect of the addition of the material on cell proliferation. The results showed that the growth trend of cells in BSAP solutions were comparable to those in the original medium (Figures 5A, B). As shown in Supplementary Figure S3, the AML12 cells maintained high viability in BSAP-contained culture medium condition. In addition, the AML12 cells were also able to adhesion on BSAP hydrogel at a state of high viability (Supplementary Figure S3). These results demonstrated the good cytocompatibility of the BSAP hydrogel. To determine whether the peptide induced organs toxicity of the host, we collected the major organs and plasma of the rats on day 3 after treatment with BSAP hydrogel in the full-thickness skin wounds infection model for histological evaluation and biochemistry test. As the results shown in Figures 5C–E, there was no significant changes of organ pathology, blood routine results and biochemical indexes in BSAP treatment group compared with control group, suggesting that there was no risk of general or acute toxicity on organ and blood. In brief, all results showed that the composite BSAP hydrogel possess adequate biocompatibility.

## 4 Conclusion

In summary, we designed a novel self-assembling peptide hydrogel with favorable biosecurity, rapid hemostasis and antibacterial property. ERC and CBC peptide could rapidly self-assemble into tightly entangled nanofibers via the catalysis of Ca^2+^, which was responsible for the hydrogelation. BSAP hydrogel exhibits the shear-thinning behavior and the shear-resilience with injectability. After the injection, due to the properties of blood-clotting, red blood cells and platelets adhesion, the BASP hydrogel exhibited excellent hemostatic effect in rat foot trauma model and rat tail amputation model. Furthermore, the remarkably antibacterial ability of BSAP hydrogel was also confirmed, which is helpful to accelerate the wound-healing. What’s more, the ERC and CBC peptide exhibited the low toxicity against normal mammalian cells and organs as well as decreased the inflammatory response of wound. Collectively, this work provided an effective strategy to develop a multifunctional self-assembling peptide hydrogel with hemostasis and antibacterial property exhibiting excellent potential for clinical application.

## Data Availability

The original contributions presented in the study are included in the article/Supplementary Material further inquiries can be directed to the corresponding authors.
