# Supplementary material for: A Bionic Self-Assembly Hydrogel Constructed by Peptides With Favorable Biosecurity, Rapid Hemostasis and Antibacterial Property for Wound Healing
Source: Front Bioeng Biotechnol. 2022 Jun 30;10:901534. doi: 10.3389/fbioe.2022.901534 (PMC9279901; doi:10.3389/fbioe.2022.901534)
Supplement: Supplementary file 1 [file DataSheet1.docx]

Supplementary Material

A bionic self-assembly hydrogel constructed by peptides with favorable biosecurity, rapid hemostasis and antibacterial property for wound healing

**Yang Wang^1,2,^** ^†^**, Xiao Li^3,^** ^†^**, Juzheng Yuan^3^****^, †^, Xudan Wang^3^, Kaishan Tao^3*^, Jin Yan^1,2*^**

^1^National & Local Joint Engineering Research Center of Biodiagnosis and Biotherapy, The Second Affiliated Hospital of Xi'an Jiaotong University, Xi'an, China.

^2^Department of Tumor and Immunology in precision medical institute, Western China Science and Technology Innovation Port, Xi'an, China.

^3^Department of Hepatobiliary Surgery, Xijing Hospital, The Fourth Military Medical University, Xi'an, China

***Correspondence:**Jin Yan
yanjin19920602@xjtu.edu.cn
Kaishan Tao
[taokaishan0686@163.com](mailto:taokaishan0686@163.com)

^†^ These authors have contributed equally to this work

**Supplementary Figure**

**
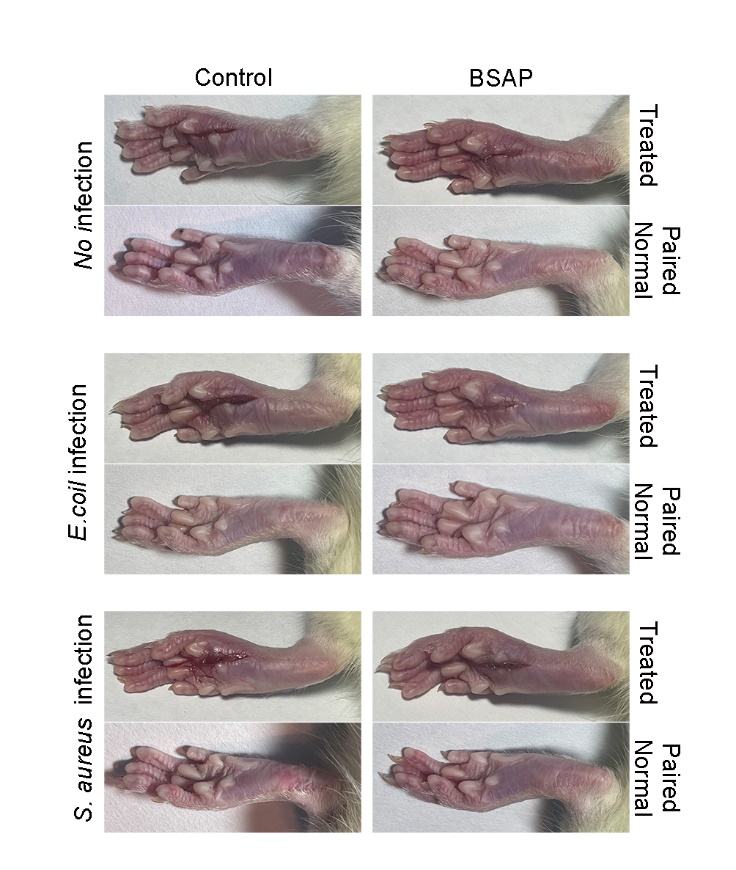
**

**Figure S1**. Representative wound photos of rat foot trauma model infected by *E. coli* or *S. aureus* and treated with BSAP hydrogels. “Paired normal” means the left foot without any treatment corresponding to the operated and treated right foot in one rat.


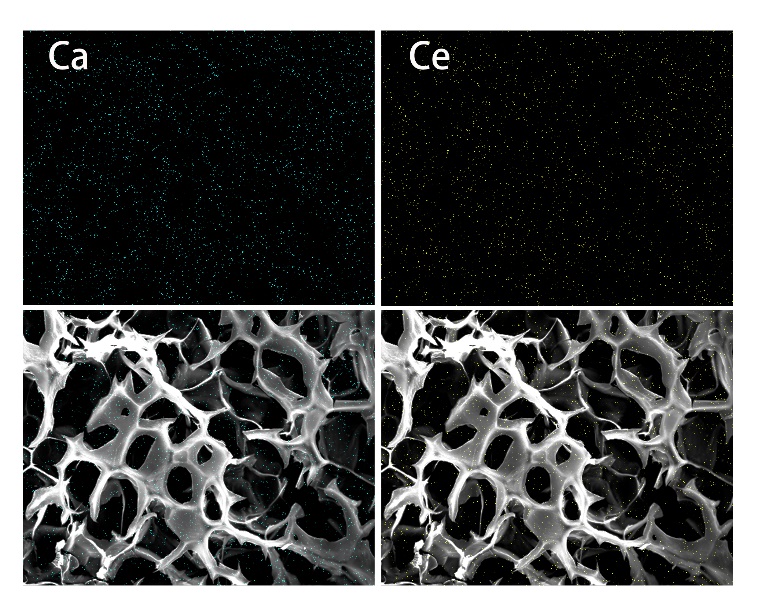


**Figure S2**. SEM image and elemental (Ca and Ce) analysis of BSAP hydrogel.


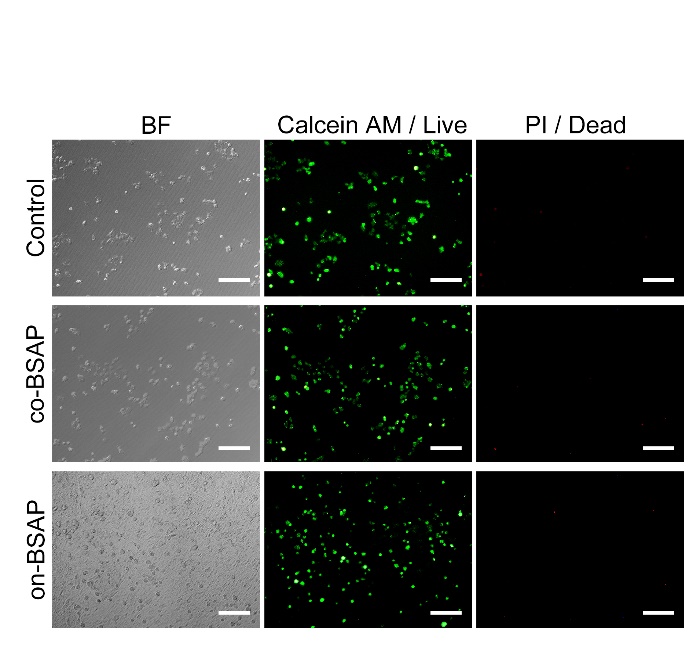


**Figure S3.** Cytocompatibility of the BSAP hydrogel. Live-dead staining of AML12 cells: upper panel, cells cultured in fresh medium on dish; middle panel, cells cultured in fresh medium containing with 0.075% BSAP hydrogel on dish; lower panel, the cells cultured in fresh medium on BSAP hydrogel. BF (bright field), calcein-AM (calcein; green; live), PI (propidium iodide; red; dead). Scale bar: 200μm.


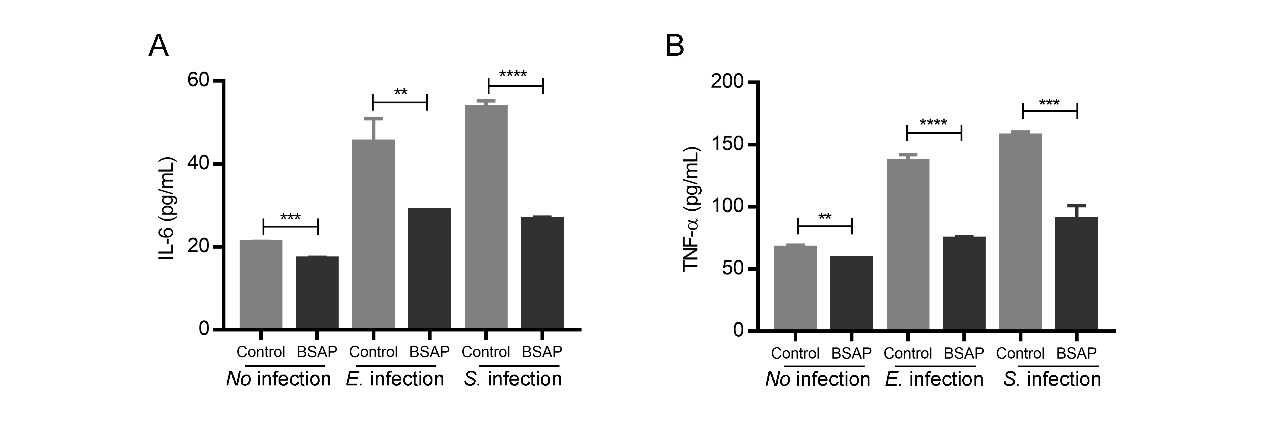


**Figure S4.** ELISA assay of two inﬂammatory factors: IL-6 and TNF-α (n=3).

**Supplementary Table**

| **Table S1. Blood routine analysis of full-thickness skin wounds infection model after one day** | | | | | | |
| --- | --- | --- | --- | --- | --- | --- |
| Indexes | Mean±SD | | | | | |
|  | Control | BSAP | EI | BSAP+EI | SI | BSAP+SI |
| WBC(10^9^/L) | 6.8±1.2 | 5.6±1.3 | 12.8±1.3 | 7.5±1.3 | 13.6±1.2 | 8.3±0.8 |
| Lymphocyte(10^9^/L) | 5.1±0.9 | 4.2±1.1 | 9.1±0.9 | 5.4±0.8 | 9.1±0.7 | 6±0.5 |
| Monocyte(10^9^/L) | 0.4±0.1 | 0.3±0 | 0.4±0.1 | 0.4±0.1 | 0.6±0.1 | 0.4±0.1 |
| Granulocyte(10^9^/L) | 1.3±0.1 | 1±0.2 | 3.3±0.4 | 1.7±0.4 | 3.9±0.5 | 1.9±0.2 |
| Lymph(%) | 74.8±1 | 75.6±2.5 | 70.7±0.8 | 72.7±1.6 | 67.1±1.1 | 72.3±1.2 |
| Mon(%) | 5.9±0.4 | 5.7±1.1 | 3.3±0.6 | 5.3±0.3 | 4.2±0.5 | 5.2±0.7 |
| Gran(%) | 19.3±1.3 | 18.7±1.8 | 26±0.2 | 22±1.3 | 28.8±0.8 | 22.6±0.8 |
| RBC(10^12^/L) | 7.9±0.6 | 7.9±0.4 | 7.9±0.4 | 7.9±0.3 | 7.8±0.6 | 7.8±0.1 |
| HGB(g/L) | 143±6.2 | 143±3.6 | 143.7±2.4 | 144.3±2.5 | 143±6.2 | 147±2.9 |
| HCT(%) | 42.5±1.6 | 42.6±1.1 | 42.7±0.3 | 42.6±1.5 | 42.5±0.5 | 42.5±1.7 |
| MCV(fL) | 53.9±2.2 | 54±2.2 | 54.3±3 | 53.9±1.1 | 53.7±2.9 | 54±1.4 |
| MCH(pg) | 18.5±0.4 | 18.4±0.1 | 18.5±0 | 18.5±0.6 | 18.4±0.4 | 18.5±0 |
| RDW(%) | 15.7±1.2 | 15.7±0.6 | 15.7±0.5 | 16±0.9 | 15.7±1.3 | 15.3±0.6 |
| PLT(10^9^/L) | 999.5±122.1 | 1100.5±81.3 | 999.5±73.9 | 1099.5±163.7 | 999.5±74 | 1081.5±154.6 |
| *Note: EI means the E. coli infection. SI means the S. aureus infection.* | | | | | | |
